# Comparison of cervical muscle isometric force between migraine subgroups or migraine-associated neck pain: a controlled study

**DOI:** 10.1038/s41598-021-95078-4

**Published:** 2021-07-29

**Authors:** Lidiane Lima Florencio, Anamaria Siriani de Oliveira, Carina Ferreira Pinheiro, Tenysson Will-Lemos, Fabíola Dach, César Fernández-de-las-Peñas, Débora Bevilaqua-Grossi

**Affiliations:** 1 Department of Physical Therapy, Occupational Therapy, Rehabilitation and Physical Medicine, Universidad Rey Juan Carlos, 28922 Madri, Spain; 2 Department of Health Sciences, Ribeirão Preto Medical School of the University of São Paulo, Ribeirão Preto, 14049-900 Brazil; 3 Department of Neurosciences and Behavioral Sciences, Ribeirão Preto Medical School of the University of São Paulo, Ribeirão Preto, 14049-900 Brazil

**Keywords:** Neurology, Signs and symptoms

## Abstract

This study aimed to verify if migraine frequency or migraine-associated neck pain were associated with a reduction of normalized force and altered electromyographic activity during maximal cervical muscle isometric contractions. Additionally, it aimed to assess the correlation of normalized isometric force with years with migraine, headache frequency, headache intensity, migraine-related disability, and severity of cutaneous allodynia. The sample comprises 71 women with migraine (40/31 episodic/chronic, 42/18 with/without neck pain) and 32 women without headache. Cervical muscle isometric force in flexion, extension, and lateral flexion was assessed synchronized with the acquisition of superficial electromyography from the cervical muscles. Women with episodic migraine presented lower normalized isometric force in extension, flexion, and right and left lateral flexions than controls (*P* < 0.05). Women with migraine and neck pain exhibited lower cervical extension and right/left lateral-flexions normalized isometric force than controls (*P* < 0.05). No significant differences were observed in antagonist activity. Normalized isometric force in all directions showed weak to moderate correlations with the severity of self-reported symptoms of cutaneous allodynia (− 0.25 ≥ r ≥ − 0.39). No additional linear correlation with clinical migraine features was observed. In conclusion, cervical muscle weakness may be associated with episodic migraine and neck pain concurrent with migraine attacks without altered antagonist activity. Additionally, it may also be related to the severity of cutaneous allodynia.

## Introduction

Migraine is a recognized disabling and chronic condition that affects general population^1,2^. It is a primary headache characterized by recurrent attacks of severe, pulsating, or unilateral headaches associated with other central sensitization components such as photophobia, phonophobia, and nausea^3,4^. Recent literature reinforces the need to consider migraine subgroups to determine proper clinical strategies or to optimize the knowledge of each phenotype because of the wide variety of clinical manifestations^4–7^.

The most commonly used grouping strategy of migraine is based on the frequency of attacks. It classifies migraine as episodic or chronic, according to the general cut-off of 15 days with headaches per month^3^. Patients with chronic migraine present more related disability, alterations in the central nervous system function and structure, and distinct treatment response than those with episodic migraine^8^. Nonetheless, patients with chronic migraine also seem to exhibit fewer associated symptoms and less severe pain^8^.

It is also recognized that neck pain is highly prevalent in individuals with migraine^9–13^ and is associated with a more severe migraine-related disability, a worse prognosis to pharmacological treatment, and a higher frequency of attacks^5,14–17^. Considering the role of physiotherapists in the multidisciplinary management of people with migraine^18–21^ a better characterization of functional disorders involving the neck pain concurrently with migraine would be of interest to practitioners.

There is evidence supporting the presence of cervical musculoskeletal dysfunctions in migraine^22–24^; however, the evidence is conflicting in some aspects. A meta-analysis^22^ showed a very low association between migraine and muscle strength and altered superficial muscle activity during isometric contractions. However, other meta-analysis^24^ indicated lower strength for extensors in patients with migraine compared to controls. Both systematic reviews^22,24^ cited three common points: (1) more studies are needed, (2) the discrimination of the presence/absence of neck pain in migraine groups, and (3) the potential influence of chronicity (episodic/chronic).

Few studies have discriminated the presence/absence of migraine-related neck pain and the attack frequency when assessing cervical musculoskeletal dysfunctions in patients with migraine^10,25–28^. For the cervical muscle isometric force, only the frequency has been considered. Reduced cervical extensor force was found in the chronic migraine group, even though both episodic and chronic groups presented increased antagonist activity during maximal cervical contractions^27^.

Therefore, we aimed to investigate the differences in muscle isometric force and electromyographic activity among (1) women with episodic migraine, chronic migraine, and controls without headache, (2) women with migraine without neck pain, migraine and neck pain, and controls without headache and neck pain. Additionally, we aimed to assess the correlation between pain and the reported impact of migraine and cervical muscle force and activity in women with migraine.

## Methods

### Participants

**Figure 1:**
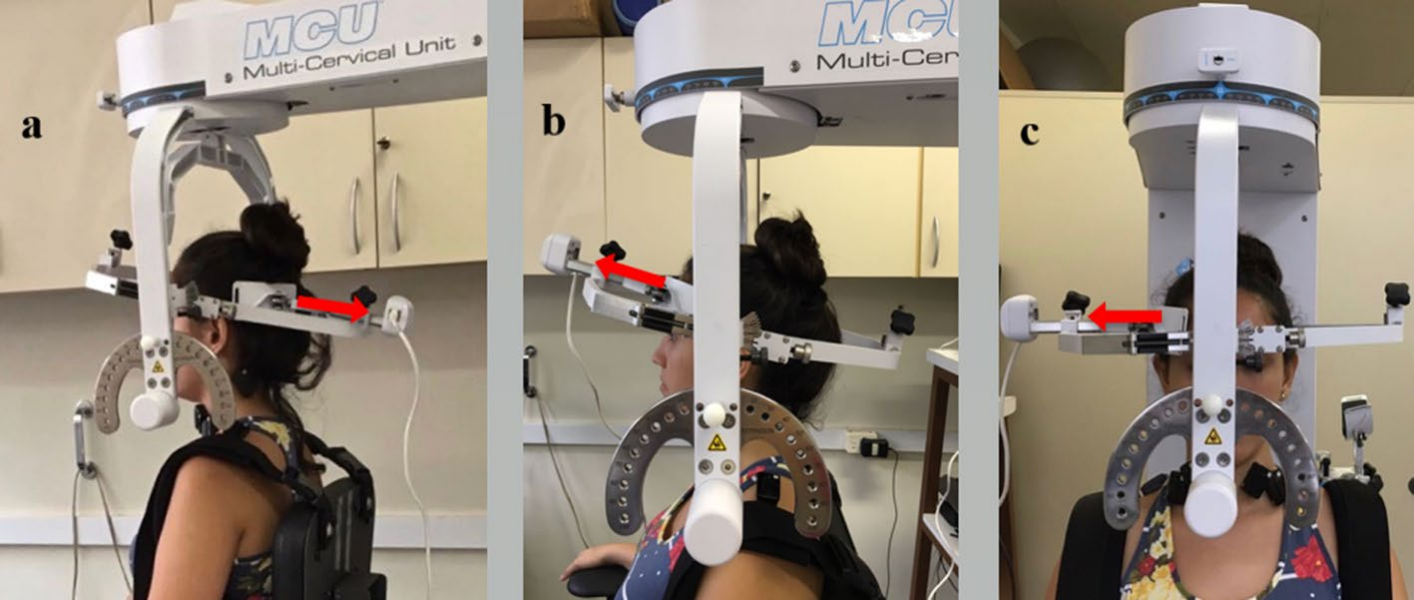
Representation of a participant during strength measurement of cervical extensors (**a**), flexors (**b**) and lateral flexion (**c**) using the Multi-Cervical Rehabilitation Unit (MCU).

From January 2018 to August 2019, women aged 18 and 55 years were consecutively recruited by social media advertisements among the local community. An experienced neurologist performed a migraine diagnosis according to the third edition of the International Headache Society criteria^3^. They were excluded if they presented with: other concomitant headaches diagnosis, cervical-related pathology or degenerative cervical conditions, history of neck or head trauma, previous anesthetic nerve block treatment, or had received physical therapy the previous year for migraine or pregnancy. For the control group, participants had to report no previous history of headache. Also, for the second objective based on the grouping approach considering the presence of neck pain, they also had to present no history of neck pain symptoms the previous year. The protocol of this study was approved by Ethics Committee in Research from the Ribeirão Preto Medical School of the University of São Paulo, Brazil (Process Number 12145/2016), which guarantee that all methods were performed following international guidelines as the Helsinki Declaration and the International Ethical Guidelines for Biomedical Research Involving Human Subjects—CIOMS/WHO (Brazilian National Health Council Resolution 466/12 and supplement). All participants provided written informed consent, and their rights were protected. Also, informed consent was obtained to publish the individual’s image that appears in Fig. 1 in an online open access publication.

Volunteers were interviewed about the presence of self-reported neck pain and pain perception. The migraine group was also questioned if they associated their neck pain with migraine attacks (prodrome, headache phase, or postdrome). Migraine characteristics included the frequency (headache days/month), intensity (numerical pain rate scale [NPRS], 0–10), and years with migraine. Migraine-related disability was measured using the MIDAS questionnaire^29^, whereas the presence and severity of cutaneous allodynia during migraine attacks were assessed using the ASC-12^30^.

Patients were grouped by frequency of headache (episodic or chronic migraine) and concomitant (presence/absence of) migraine-associated neck pain. Frequency-based grouping included the control group without a history of headache; chronic migraine group, with 15 or more days with headache per month, for more than three months, which, on at least eight days/month, has the features of migraine headache^3^; and episodic migraine, with 1–12 days of headache per month, for more than three months.

The grouping approach considering neck pain was performed independently of the migraine frequency subgroup (episodic or chronic migraine). Participants were stratified among a control group without a history of headache or neck pain, a group with migraine without neck pain, and a group with migraine and related neck pain. Neck pain related to migraine attacks was identified by asking specifically to the participants if they perceived their neck pain to be related to their headache attacks (before, during, or after them). We just included in this group those who relate their neck pain to migraine attacks; participants with migraine who related their neck pain with other reasons, such as labor postures or stress, were excluded from this analysis.

### Cervical muscle normalized isometric force

An examiner blinded to the subject's condition assessed the cervical muscle isometric force with participants in a pain-free period (neither headache nor neck pain). It was measured with participants in a sitting position using the Multi-Cervical Rehabilitation Unit (MCU) (Baltimore Therapeutic Equipment Technologies, Hanover, MD, USA). The MCU is a fixed frame load cell customized to assess the cervical spine with excellent reliability (ICC 0.92–0.99) for cervical muscle force assessment^31^. The system was calibrated daily following the manufacturer's guidance.

Data was obtained from maximal isometric voluntary contractions (MIVC) in extension, flexion, and lateral flexion of the cervical spine. Participants were seated, fixed firmly with the MCU belts to stabilize the trunk, with the head and neck in a neutral position, aligned with the axis of the MCU system due to adjustments in the seat height. The load cell was positioned at the occiput protuberance to assess the isometric force of the extensor muscles (Fig. 1a), at the upper portion of the participant's eyebrows and the midline of the frontal bone to test the cervical flexors (Fig. 1b), and at the temporal bone, 2 cm above the ear (Fig. 1c) to test cervical lateral flexion.

Participants were familiarized with the test positions by performing a submaximal contraction. Then, three repetitions for each direction were performed, randomly chosen by drawing. Participants sustained the maximal isometric voluntary contractions for 3 s, with a 15 s rest-period between repetitions and a 2 min rest period between directions. The examiner used standardized verbal encouragement during each assessment.

### Electromyographic acquisition and data management

Electrical activity using electromyography (EMG) from the sternocleidomastoid, anterior scalene, splenius capitis, and upper trapezius was acquired simultaneously using the TrignoTM Wireless System (CMRR of 80 dB, input impedance exceeding 1000 Ω, Delsys Inc. Boston, MA, USA) sampled at 4 kHz. The Trigno sensor was firmly fixed with adhesive tape bilaterally after proper skin cleaning according to standard instructions for electrode placement. Electrodes were placed according to standard instructions^32–34^.

Acquisition of the MCU and EMG signals were synchronized using the Transistor-Transistor Logic Trigger Module (Delsys Trigno) and an A/D converter board (USB-1616HS-BNC; Measurement Computing Corporation, Norton, MA, USA). Both the Trigno™ Wireless System and the A/D converter board were connected to an external power supply to avoid power grid noise. The MCU and EMG data were relayed to a customized MATLAB script and sampled at 2 kHz. Data were analyzed using a custom MATLAB code (MathWorks, Natick, MA, USA). The peak force of each trial was provided, and for comparison purposes, it was converted into Newtons (N) and normalized by the participant's mass [(MCU data (kgF) × 9.81)/body mass (Kg)].

The EMG’s raw signals were band-filtered at 20–500 Hz (4th order Butterworth), and the average root-mean-square was calculated from the central 2 s window. Antagonist muscle activity was normalized by its respective activity obtained from the trial when acting as an agonist (% MIVC)^27^.

### Statistical analysis

Statistical analysis was performed using SPSS version 20.0 (IBM Corporation, Armonk, NY, USA), adopting a significance level of 0.05. Parametric tests were applied when the normal distribution of the residuals could be confirmed by the Shapiro–Wilk test, even when the logarithmic transformation was needed. Non-parametric tests were applied when normal distribution could not be confirmed. Sample characteristics comparison among groups was conducted using a one-way analysis of variance (ANOVA) with Bonferroni’s test as a post hoc analysis for each grouping strategy.

Separate one-way ANOVAs were performed to compare isometric muscle normalized force on each cervical direction (extension, flexion, and right and left lateral flexion). To address the primary objective, the between-group factor was the migraine type (episodic migraine, chronic migraine, and control group) and; to address the secondary objective, the between-group factor was the presence/absence of neck pain related to migraine attacks (migraine without neck pain, migraine with neck pain and control group). Antagonist activity produced during MIVC was compared by the Kruskal–Wallis test using the same grouping approach defined by the chronicity of migraine episodes and by the presence of neck pain. Finally, Spearman's correlation coefficient was calculated to assess the potential correlation between the cervical muscle normalized isometric force and years with migraine, headache frequency, headache intensity, migraine-related disability, and severity of cutaneous allodynia. Correlations were classified as weak (rho < 0.30), moderate (rho between 0.30 and 0.70), and strong (rho > 0.70)^35^.

## Results

**Figure 2:**
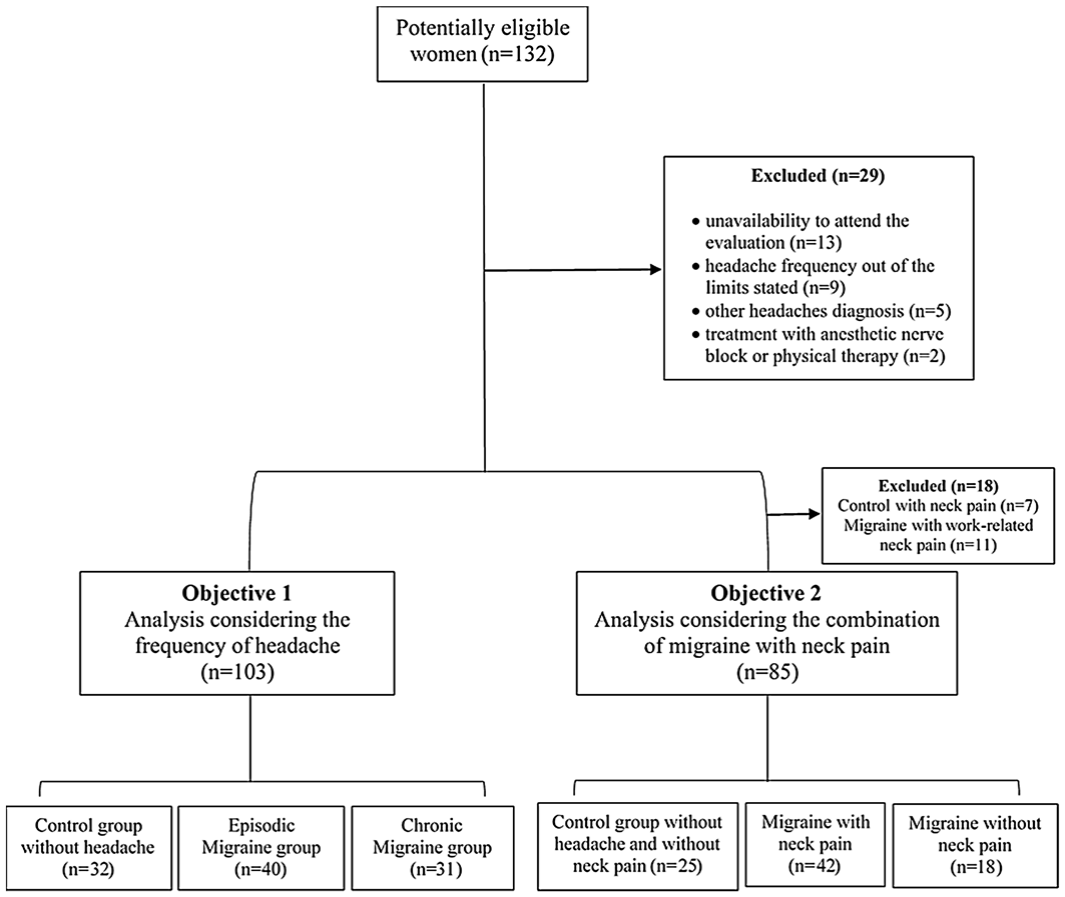
Flow diagram of the participant's recruitment and inclusion.

A flow diagram describing the recruitment procedures used to achieve the final sample and the reasons for exclusion is presented in Fig. 2. The sample characteristics are presented in Table 1. There were no differences among groups in terms of age and body mass index. Episodic and chronic migraines differed only in headache frequency, as expected by their definition (*P* < 0.001). Patients with migraine and related neck pain exhibited worse migraine-related disability and greater severity of cutaneous allodynia than those with migraine but without neck pain (*P* < 0.05).

**Table 1:** Characteristics of the sample.

	Frequency grouping approach			Neck pain grouping approach^a^		
	Control (n = 32)	Episodic migraine (n = 40)	Chronic migraine (n = 31)	Control (n = 25)	Migraine with neck pain (n = 42)	Migraine without neck pain (n = 18)
Age	31.5 (9.3)	32.5 (8.8)	34.6 (9.9)	31.7 (9.8)	34.8 (9.1)	31.3 (9.7)
Body mass Index (kg/cm^2^)	24.9 (4.1)	24.2 (4.1)	23.7 (2.9)	25.0 (4.4)	24.1 (3.4)	24.1 (4.3)
**Migraine related characteristics**						
Years with migraine		14.4 (8.3)	17.6 (10.9)		15.2 (8.9)	16.9 (11.6)
Headache frequency (days/month)		6.8 (3.2)	24.2 (5.6)*		16.4 (9.9)	12.4 (10.1)
Headache intensity (0–10) [NPRS]		7.6 (1.5)	8.1 (1.7)		7.8 (1.7)	7.9 (1.6)
MIDAS		49.9 (37.5)	69.9 (58.0)		68.7 (52.7)	31.8 (23.2)**
ASC-12		9.2 (4.4)	7.8 (3.9)		9.4 (3.6)	5.6 (4.1)**

^a^11 subjects were excluded from this analysis due to work-related neck pain.

*NPRS* numeric pain rating scale, *MIDAS* migraine-related disability questionnaire, *ASC-12* 12 item Allodynia Symptom Checklist.

*Different from episodic migraine (*P* < 0.001); **different from migraine with neck pain group (*P* < 0.05).

### Cervical muscle normalized isometric force

When grouping patients with migraine based on the headache frequency, significant differences were found between episodic migraine and controls: women with episodic migraine had lower normalized isometric force than controls (flexion: Δ − 0.25 N/Kg; 95% CI − 0.49 to − 0.01; extension: Δ − 0.42 N/Kg; 95% CI − 0.77 to − 0.07; left lateral-flexion: Δ − 0. 28 N/Kg; 95% CI − 0.51 to − 0.06; right lateral flexion: Δ − 0.26 N/Kg; 95% CI − 0.51 to − 0.02). However, chronic migraine group did not differ from controls, and no differences between the episodic and chronic migraine subgroups were observed (Table 2).

**Table 2:** Cervical muscle strength data [mean (standard deviation)] for the migraine frequency-based grouping approach.

	Controls (n = 32)	Episodic migraine (n = 40)	Chronic migraine (n = 31)	ANOVA
**Extension**				
Normalized force (N/kg)	1.94 (0.71)	1.52 (0.57)*	1.70 (0.54)	F = 4.348; *P* = 0.02
Force (N)	121.98 (39.41)	97.50 (35.15)	106.52 (34.92)	
**Flexion**				
Normalized force (N/kg)	1.19 (0.46)	0.95 (0.36)*	1.06 (0.43)	F = 3.127; *P* = 0.048
Force (N)	75.6 (27.43)	60.90 (23.83)	65.75 (25.26)	
**RLF**^**a**^				
Normalized force (N/kg)	1.29 (0.53)	1.02 (0.41)*	1.06 (0.30)	F = 3.652; *P* = 0.03
Force (N)	81.22 (29.88)	66.16 (26.61)	66.36 (19.16)	
**LLF**				
Normalized force (N/kg)	1.30 (0.47)	1.01 (0.38)*	1.09 (0.32)	F = 4.787; *P* = 0.01
Force (N)	82.66 (30.17)	65.11 (23.52)	68.84 (21.40)	

*RLF* right lateral flexion, *LLF* left lateral flexion.

^a^Log-transformed variable.

*P < 0.05, different from the control group in post hoc comparisons.

When grouping patients with migraine by the presence/absence of related neck pain, the migraine group with neck pain presented a lower normalized isometric force than controls (extension: Δ − 0.39 N/Kg; 95% CI − 0.76 to − 0.01; left lateral flexion: Δ − 0.30 N/Kg; 95% CI − 0.54 to − 0.05; right lateral flexion: Δ − 0.33 N/kg; 95% CI − 0.59 to − 0.06). No significant differences were observed between migraine without neck pain and controls, neither between migraines with and without neck pain subgroups (Table 3).

**Table 3:** Cervical muscle strength data [mean (standard deviation)] for neck pain grouping approach.

	Controls (n = 25)	Migraine with neck pain (n = 42)	Migraine without neck pain (n = 18)	ANOVA
**Extension**				
Normalized force (N/kg)	1.92 (0.73)	1.53 (0.52)*	1.66 (0.60)	F = 3.206; *P* = 0.046
Force (N)	119.0 (37.1)	97.9 (34.5)	103.7 (35.5)	
**Flexion**				
Normalized force (N/kg)	1.21 (0.44)	0.98 (0.41)	1.04 (0.41)	F = 2.356; *P* = 0.10
Force (N)	75.3 (23.9)	61.6 (24.4)	65.4 (26.8)	
**RLF**^**a**^				
Normalized force (N/kg)	1.31 (0.58)	0.99 (0.36)*	1.16 (0.35)	F = 4.529; *P* = 0.01
Force (N)	81.2 (28.1)	63.2 (24.7)	72.4 (20.3)	
**LLF**				
Normalized force (N/kg)	1.30 (0.50)	1.01 (0.36)*	1.18 (0.31)	F = 4.656; *P* = 0.01
Force (N)	81.4 (27.6)	64.2 (24.2)	73.9 (17.6)	

*RLF* right lateral flexion, *LLF* left lateral flexion.

^a^Log-transformed variable.

*P < 0.05, different from control group in post hoc analysis.

### Antagonist muscle activation

**Figure 3:**
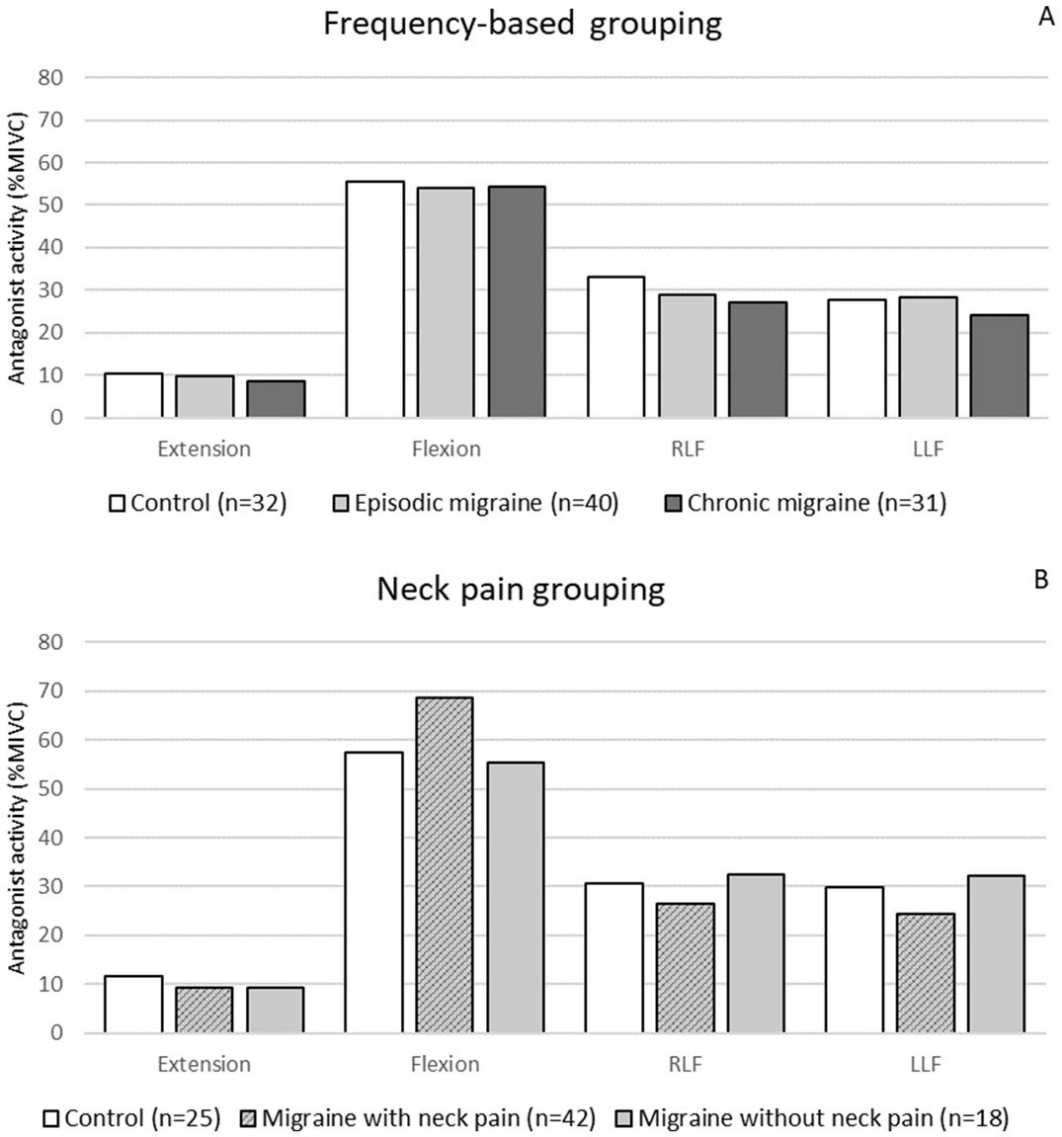
Antagonist activity during maximal isometric voluntary contractions (MIVC) in cervical extension, flexion, right lateral flexion (RLF), and left lateral flexion (LLF). Data were compared stratifying migraine groups as episodic and chronic migraine (**A**) or migraine with and without neck pain (**B**).

The antagonist activity during MIVC is shown in Fig. 3. No significant differences were observed among episodic migraine, chronic migraine, and controls for extension (H = 0.226; *P* = 0.89), flexion (H = 0.540; *P* = 0.76), right (H = 2.443; *P* = 0.30), and left (H = 0.626; *P* = 0.73) lateral-flexions (Fig. 3A). Similarly, no significant differences were observed among controls, migraine with related-neck pain and migraine without neck pain for any movement: extension (H = 1.444; *P* = 0.49), flexion (H = 4.969; *P* = 0.08), and right (H = 1.011; *P* = 0.60) and left lateral flexion (H = 2.186; *P* = 0.34) (Fig. 3B).

### Association with migraine clinical characteristics

Weak to moderate significant negative correlations were observed between 12-item allodynia symptom checklist (ASC-12) score and the neck muscle normalized isometric force in flexion (rho: − 0.31; *P* = 0.01), extension (rho: − 0.35; *P* = 0.003), and right (rho: − 0.25; *P* = 0.03) and left (rho: − 0.39; *P* = 0.001) lateral flexion. No other significant correlation was found between normalized isometric force in all directions and years with migraine, headache frequency, headache intensity, Migraine Disability Assessment (MIDAS) questionnaire, and ASC-12 scores.

## Discussion

In the current study, women with episodic migraine and women with migraine and associated neck pain presented lower normalized isometric force than controls. However, no differences were found in the amplitude of neck muscle antagonist activation. Finally, the cervical muscle normalized isometric force presented a weak to moderate correlation with cutaneous allodynia severity but not with other migraine clinical characteristics.

The frequency-based grouping strategy revealed that only women with episodic migraine showed lower cervical normalized isometric forces, contrary to previous studies reporting reduced cervical extensor normalized isometric force in women with chronic (not episodic) migraine^27^. Other studies have reported weak negative correlations between cervical muscle strength and headache frequency in patients with migraine^27,36^. Our findings did not show any significant correlation between cervical normalized isometric forces and the frequency of headache. Despite the usefulness of frequency-based grouping for migraine-related disability or treatment prognosis^8^, it may not be as relevant when dealing with cervical muscle normalized isometric forces. This hypothesis is supported by a recent study showing that no cluster of musculoskeletal cervical spine impairments, including joint, sensory, and proprioceptive aspects, could differentiate between episodic and chronic migraine^7^.

Our study is the first to report that lower cervical muscle force is confirmed when both migraine and related neck pain coexist. It agrees with other studies showing that patients with migraine and related-neck pain present greater neck muscle tenderness^37,38^, greater reduced upper cervical mobility, and worse performance in the craniocervical flexion test^10^ than patients with migraine only. Hvedstrup et al.^38^ demonstrated that both migraine with and without ictal neck pain presented greater neck muscle tenderness than controls without headache, but those with ictal neck pain are even more sensitized. Herein, only the migraine group with neck pain presented reduced muscle force. It suggests that the association of migraine and ictal neck pain may be an aggravating factor to local tenderness but may be a predisposing factor to musculoskeletal impairment such as lower force. As the remaining previous studies did not include a control group without migraine to contrast their data^10,37^, information to discuss whether or not the neck pain aggravate or predispose some signs and symptoms of cervical disorders is still scarce.

Therefore, future studies may consider adding a control group without migraine to compare the upper cervical mobility, the results of the craniocervical flexion test, or other variables to assess whether the co-morbidity of these two conditions (migraine and neck pain) has a greater impact on these variables rather than just the presence of migraine.

The proportion of women with migraine-related neck pain in our study was 70%, and non-related pain like labor-related neck pain was excluded. This proportion is similar to that previously reported elsewhere^9,10,13^. Our findings suggest that this particular subgroup may present not only reduced neck muscles force but also greater migraine-related disability and more severe cutaneous allodynia symptoms than women with migraine only. Therefore, clinicians may consider referring these patients for physical therapy assessment and tailored interventions.

The relationship between migraine and cervical musculoskeletal dysfunction is a controversial issue. The most accepted hypothesis providing neurological plausibility of head and neck interaction is the convergence of cervical and trigeminal afferents into the trigeminocervical nucleus caudalis^16^. The origin or cause of migraine-related neck pain is under debate involving theories based on its relationship with cervical musculoskeletal dysfunction or being an integral feature of migraine^2,23,37–41^. Therefore, the reduced force of the cervical muscles observed in our study within the migraine and neck pain group should not be interpreted merely as a neuromuscular inhibition response to local musculoskeletal pain since no causality can be assumed in our study design.

Discrepancies between studies could also be related to outcome methodology. Most previous studies have investigated muscle strength in patients with migraine in supine or prone position^27,42–44^ or did not describe the participants’ position^45^. The only study that measured cervical muscle strength in a sitting position reported no differences between adolescents with migraine and control participants^46^. Different test positioning may lead to distinct findings considering some biomechanical factors^47,48^. An antigravity position may demand greater muscle activity than the sitting position considering the head load. Indeed, no difference in the antagonist activity was observed in the sitting position in the current study. In contrast, greater antagonist activity was observed for both migraine groups, episodic and chronic, during a functional task such as the supine's craniocervical flexion^27^. Moreover, different muscle lengths from the seated to the lying position could also influence force generation. The lower extensor strength described in chronic migraine was assessed in a study using the prone position^27^, while the current differences observed only for episodic migraine in all directions were assessed in a sitting position.

While the influence of headache frequency on cervical muscle strength is not well described in the literature, our results could have an interesting clinical practice implication. When planning exercises targeting the cervical spine in patients with migraine, therapists should consider the patient's position. This is because when they are performed in a supine or prone position, greater antagonist muscle co-activation could be expected. However, if cervical muscles are trained in sitting, it would be possible to obtain less antagonist co-activation. Conversely, if exercise aims to address the agonist weakness observed in migraine specifically, exercises in sitting may benefit patients preferentially with the episodic form, while exercises performed in the supine or prone position may benefit preferentially those with the chronic form. Future studies should investigate the effects of different exercise positions on the management of migraine.

Finally, the observed significant correlation suggests that the greater the severity of cutaneous allodynia, the lower the cervical normalized isometric force output. It is important to note that the episodic migraine group exhibited greater scores on the ASC-12, which may be a potential factor that could influence between-group differences observed for cervical muscle strength. However, this difference was not significantly different, and the linear association between cutaneous allodynia and muscle strength was not very strong, with a correlation coefficient lower than 0.40.

The current study presents some limitations. Our findings are sex-specific since only women were included. We did not perform a priori sample size calculation due to the lack of data of cervical muscle force using the MCU in patients with migraine. For this reason, we may have been underpowered to conduct multivariate analysis, and our study should be considered as a hypothesis-generating. Additionally, neck pain characterization was based on a self-reported association with migraine attacks. Still, it was not explicitly registered if it was part of the prodrome phase, an associated symptom of the migraine headache phase post-prodrome. This information would have been difficult for participants to recall since they were in a headache-free phase. Future studies may consider questioning these specific details^37^ or applying a headache diary to avoid recall bias. We tried to attenuate this by excluding participants who did not relate their neck pain to migraine attacks.

Another limitation of our sample was that we excluded those patients with migraine and other headache diagnoses (such as medication-overuse headache or tension type headache), limiting the generalization as combined headaches are commonly seen in clinical practice. It should be noted that by following the third edition of the International Headache^3^, it is accepted within the diagnoses of chronic migraine that, at some days, the headache may not present the criteria for a migraine headache. However, it is mandatory to present headaches meeting migraine criteria on at least eight days per month^3^. Herein, we assessed the headache frequency without differentiating how many presented migraine criteria. As we did not observe any significant difference related to the chronic migraine group neither a significant correlation with the headache frequency, we believe that it would not influence current findings. However, future studies may benefit from a headache diary to register and consider these characteristics.

Despite its limitations, our study provides new insights into the relationship between cervical muscle impairments and migraine. Reduced cervical muscle normalized isometric force may be observed in women with episodic migraine or in women who present neck pain associated with their migraine attack. However, this impairment of cervical muscle force production does not seem to be accompanied by an altered antagonist activity. Additionally, neck muscle normalized isometric force is correlated to the severity of cutaneous allodynia, suggesting that the greater the severity of cutaneous allodynia, the lower is the expected force.

## References

[1] Burch RC, Buse DC, Lipton RB (2019). Migraine: Epidemiology, burden, and comorbidity. Neurol. Clin..

[2] Steiner TJ, Stovener LJ, Vos T (2016). GBD2015: Migraine is the third cause of disability in under 50s. J. Headache Pain.

[3] Headache Classification Committee of the International Headache Society (IHS) (2018). The international classification of headache disorders. Cephalalgia.

[4] Schürks M, Buring JE, Kurth T (2011). Migraine features, associated symptoms and triggers: A principal component analysis in the women’s health study. Cephalalgia.

[5] Lipton RB (2018). Identifying natural subgroups of migraine based on comorbidity and concomitant condition profiles: Results of (CaMEO) study. Headache.

[6] Luedtke K, May A (2017). Stratifying migraine patients based on dynamic pain provocation over the upper cervical spine. J. Headache Pain.

[7] Pérez-Benito FJ (2020). Subgrouping factors influencing migraine intensity in women: A semi-automatic methodology based on machine learning and information geometry. Pain Pract..

[8] Aurora SK, Brin MF (2017). Chronic migraine: An update on physiology, imaging, and the mechanism of action of two available pharmacologic therapies. Headache.

[9] Ashina S (2015). Prevalence of neck pain in migraine and tension-type headache: A population study. Cephalalgia.

[10] Bragatto MM (2019). Is the presence of neck pain associated with more severe clinical presentation in patients with migraine? A cross-sectional study. Cephalalgia.

[11] Calhoun AH (2010). The prevalence of neck pain in migraine. Headache.

[12] Fernández-de-las-Peñas C (2010). Population-based study of migraine in Spanish adults: Relation to socio-demographic factors, lifestyle and co-morbidity with other conditions. J. Headache Pain.

[13] Lampl C, Rudolph M, Deligianni CI, Deligianni CI, Mitsikostas DD (2015). Neck pain in episodic migraine: Premonitory symptom or part of the attack ?. J. Headache Pain.

[14] Calhoun AH, Ford S, Pruitt AP (2011). Presence of neck pain delays migraine treatment. Postgrad. Med..

[15] Carvalho GF (2014). Comparison between neck pain disability and cervical range of motion in patients with episodic and chronic migraine: A cross-sectional study. J. Manip. Physiol. Ther..

[16] Charles A (2018). The pathophysiology of migraine: Implications for clinical management. Lancet Neurol..

[17] Florencio LL (2014). Neck pain disability is related to the frequency of migraine attacks: A cross-sectional study. Headache.

[18] Castien R, de Hertogh W (2019). A neuroscience perspective of physical treatment of headache and neck pain. Front. Neurol..

[19] Gaul C, Liesering-Latta E, Schäfer B, Fritsche G, Holle D (2016). Integrated multidisciplinar care of headache disorders: A narrative review. Cephalalgia.

[20] Kristoffersen ES, Grande RB, Aaseth K, Lundqvist C, Russel MB (2012). Management of primary chronic headache in the general population: The Akershus study of chronic headache. J. Headache Pain.

[21] Sarchielli P (2012). Italian guidelines for primary headaches: 2012 revised version. J. Headache Pain.

[22] Liang Z, Galea O, Thomas L, Jull G, Treleaven J (2019). Cervical musculoskeletal impairments in migraine and tension type headache: A systematic review and meta-analysis. Musculoskelet. Sci. Pract..

[23] Luedtke K, Stark W, May A (2018). Musculoskeletal dysfunction in migraine patients. Cephalalgia.

[24] Szikszay TM (2019). Which examination tests detect differences in cervical musculoskeletal impairments in people with migraine? A systematic review and meta-analysis. Phys. Ther..

[25] Bevilaqua-Grossi D (2009). Cervical mobility in women with migraine. Headache.

[26] Ferracini GN (2017). Musculoskeletal disorders of the upper cervical spine in women with episodic or chronic migraine. Eur. J. Phys. Rehabil. Med..

[27] Florencio LL (2015). Cervical muscle strength and muscle coativation during isometric contractions in patients with migraine: A cross-sectional study. Headache.

[28] Oliveira-Souza AIS (2019). Reduced flexion rotation test in women with chronic and episodic migraine. Braz. J. Phys. Ther..

[29] Fragoso YD (2002). MIDAS (Migraine Disability Assessment): A valuable tool for work-site identification of migraine in workers in Brazil. São Paulo Med. J..

[30] Florencio LL (2012). 12 item allodynia symptom checklist/Brasil: Cross-cultural adaptation, internal consistency and reproducibility. Arq. Neuropsiquiatr..

[31] Chiu TTW, Sing KL (2002). Evaluation of cervical range of motion and isometric neck muscle strength: Reliability and validity. Clin. Rehabil..

[32] Falla D, Dall’Alba P, Rainoldi A, Merletti R, Jull G (2002). Location of innervation zones of sternocleidomastoid and scalene muscles: A basis for clinical and research electromyography applications. Clin. Neurophysiol..

[33] Joines SMB, Sommerich CM, Mirka GA, Wilson JR, Moon SD (2006). Low-level exertions of the neck musculature: A study of research methods. J. Electromyogr. Kinesiol..

[34] Surface ElectroMyoGraphy for the Non-Invasive Assessment of Muscles. http://www.seniam.org/. Accessed December (2018).

[35] Bland JM, Altman DG (1995). Calculating correlation coefficients with repeated observations: Part 2—Correlation between subjects. BMJ.

[36] Tolentino GA (2018). Relationship between headaches and neck pain characteristics with neck muscles strength. J. Manip. Physiol. Ther..

[37] Yu Z, Wang R, Ao R, Yu S (2019). Neck pain in episodic migraine: A cross-sectional study. J. Pain Res..

[38] Hvedstrup J, Kolding LT, Younis S, Ashina M, Schytz HW (2020). Ictal neck pain investigated in the interictal state: A search for the origin of pain. Cephalalgia.

[39] Jull G, Hall T (2018). Cervical musculoskeletal dysfunction in headache: How should it be defined?. Musculoskelet. Sci. Pract..

[40] Viana M (2018). When cervical pain is actually migraine: An observational study in 207 patients. Cephalalgia.

[41] Watson DH, Drummond PD (2012). Head pain referral during examination of the neck in migraine and tension-type headache. Headache.

[42] Benatto MT (2019). Extensor/flexor ratio of neck muscle strength and electromyographic activity of individuals with migraine: A cross - sectional study. Eur. Spine J..

[43] Dumas JP (2001). Physical impairments in cervicogenic headache: Traumatic vs. nontraumatic onset. Cephalalgia.

[44] Horwitz S, Stewart A (2015). An exploratory study to determine the relationship between cervical dysfunction and perimenstrual migraines. Physiother. Can..

[45] Jull G, Amiri M, Bullock-Saxton J, Darnell R, Lander C (2007). Cervical musculoskeletal impairment in frequent intermittent headache. Part 1: Subjects with single headaches. Cephalalgia.

[46] Oksanen A (2008). Force production and EMG activity of neck muscles in adolescent headache. Disabil. Rehabil..

[47] O'Leary S, Fagermoen CL, Hasegawa H, Thorsen AS, Van Wyk L (2017). Differential strength and endurance parameters of the craniocervical and cervicothoracic extensors and flexors in healthy individuals. J. Appl. Biomech..

[48] Strimpakos N (2011). The assessment of the cervical spine. Part 2: Strength and endurance/fatigue. J. Bodyw. Mov. Ther..

